# Correction: Effects of service dogs on children with ASD’s symptoms and parents’ well-being: On the importance of considering those effects with a more systemic perspective

**DOI:** 10.1371/journal.pone.0315651

**Published:** 2024-12-09

**Authors:** The PLOS ONE Staff

The authors are listed out of order. Please view the correct author order, affiliations, and citation here:

Nicolas Dollion^1^, Margot Poirier^2^, Handi’Chiens^3^, Fondation Mira^4^, Florian Auffret^3^, Nathe François^4^, Pierrich Plusquellec^5,6^, Marine Grandgeorge^2^

**1** Laboratoire C2S (Cognition Santé Société)–EA6291, Université Reims Champagne-Ardenne, Reims, France, **2** CNRS, EthoS (Éthologie animale et humaine)—UMR6552, Normandie Univ, Univ Rennes, Rennes, France, **3** Association Handi’chiens Inc., Paris, France, **4** Fondation Mira, Sainte-Madeleine, QC, Canada, **5** Centre d’études en sciences de la communication non verbale, Research Centre, Montréal Mental Health University Institute, CIUSSS Est, Montréal, Canada, **6** School of Psychoeducation, University of Montréal, Montréal, QC, Canada.

Dollion N, Poirier M, Handi’Chiens, Fondation Mira, Auffret F, François N, et al. (2024) Effects of service dogs on children with ASD’s symptoms and parents’ well-being: On the importance of considering those effects with a more systemic perspective. PLOS ONE 19(1): e0295702. https://doi.org/10.1371/journal.pone.0295702.

The publisher apologizes for the errors.
