# Supplementary material for: Effects of service dogs on children with ASD’s symptoms and parents’ well-being: On the importance of considering those effects with a more systemic perspective
Source: PLoS One. 2024 Jan 3;19(1):e0295702. doi: 10.1371/journal.pone.0295702 (PMC10763939; doi:10.1371/journal.pone.0295702)
Supplement: S1 Table — (DOCX) [file pone.0295702.s001.docx]

**Supporting Information:**

**S1 Table. Additional information relative to the standardized scales and questionnaires used in the present study.**

| **Scale /Questionnaire** | **Reference** | **Cronbach Alpha** | **Goal** | **Number of items** | **Type and modality of response** | **Extracted scores** | **Duration** | **Concerned Follow-up** |
| --- | --- | --- | --- | --- | --- | --- | --- | --- |
| **ABI-S**  (*Autism Behavior Inventory Short Form*) | Bangerter A, Ness S, Lewin D, Aman MG, Esbensen AJ, Goodwin MS, et al. Clinical validation of the Autism Behavior Inventory: Caregiver-rated assessment of core and associated symptoms of autism spectrum disorder. J Autism Dev Disord. 2020;50(6):2090–101. | 0.69–0.79 | Measure of changes in core symptoms of ASD (Social Communication and Restrictive and Repetitive Behaviors) and associated domains (Mental Health, Self-Regulation and Challenging Behavior) | 24 items | 4-points Likert scale to refer:   - how often the child displays the referred behavior - child’s degree of autonomy in the referred behavior | Total score | < 10 min. | T0-T1-T2 |
| **PSI_SF**  (*Parenting Stress Index short form*) | Abidin RR. Parenting stress index: Professional manual. Odessa, FL: Psychological Assessment Resources, Inc; 1995. | 0.80-0.91 | Measure of participant’s parenting stress through three subscales (Parent Distress, Difficult Child, and Parent-Child Dysfunctional) | 36 items | 5-points Likert scale to refer:   - participant’s degree of agreement with statement - participant’s degree of difficulty - number of child’s behaviors that bother the participant | Total score | < 10 min. | T0-T1-T2 |
| **STAI_Y**  (*State and Trait Anxiety Inventory Form Y*) | Spielberger CD, Gorsuch RL, Lushene R, Vagg PR, Jacobs GA. State-Trait Anxiety Inventory for adults: manual, instrument and scoring guide. Palo Alto: Consulting Psychologists Press.; 1983. | 0.86-0.95 | Measure of participant’s state and trait anxiety | 40 items | 4-points Likert scale to refer:   - participant’s degree of agreement with statement | Score on two subscales:   - state anxiety - trait anxiety | 10 min. | T0-T1-T2 |
| **PSDQ**  (*Parenting Styles and Dimensions Questionnaire*) | Robinson CC, Mandleco B, Olsen SF, Hart CH. Authoritative, authoritarian, and permissive parenting practices: Development of a new measure. Psychol Rep [Internet]. 1995;77(3):819–30. | 0.75-0.91 | Measure of parenting style according to three major styles (authoritative, authoritarian and permissive) themselves assessed though several subscales | 62 items | 5-points Likert scale to refer:   - how often participant displays the referred of behavior | Scores on three main subscales:   - authoritative - authoritarian - permissive | 15-20 min. | T0-T1-T2 |
| **SCQ**  (*Social Communication Questionnaire*) | Rutter, M., Bailey, A., and Lord, C. The Social Communication Questionnaire (SCQ). Torrance, Canada: Western Psychological Services; 2003 | 0.90 | Screening of communication skills and social functioning difficulties (potentially indicative of ASD) | 40 items | Yes/No questions to indicate:   - if the child display(ed) or not the referred behavior | Total score | < 10 min. | T0 |
| **MDORS**  (*Monash Dog-Owner Relationship Scale*) | Dwyer F, Bennett PC, Coleman GJ. Development of the monash dog owner relationship scale (MDORS). Anthrozoos [Internet]. 2006;19(3):243–56. | 0.67-0.84 | Measure of the quality of relationship between owner and his/her dog though three subscales (owner-dog interaction, emotional closeness and perceived costs)  *For the purpose of the present study items were reformulated in order to assess for the quality of relationship between participants’ child and the dog.* | 28 items | 5-points Likert scale to refer:   - participant’s degree of agreement with statement about their child interaction/relationship with the dog - how often the child displays the referred behavior - degree of trauma or of difficulty for the child | Scores on three dimensions:   - child-dog interaction - perceived emotional closeness - perceived costs | 5 min. | T1-T2 |
