# Supplementary material for: Effects of service dogs on children with ASD’s symptoms and parents’ well-being: On the importance of considering those effects with a more systemic perspective
Source: PLoS One. 2024 Jan 3;19(1):e0295702. doi: 10.1371/journal.pone.0295702 (PMC10763939; doi:10.1371/journal.pone.0295702)
Supplement: S2 Table — (DOCX) [file pone.0295702.s002.docx]

| **Before SD Integration (T0)** | | | | | | | | | | |
| --- | --- | --- | --- | --- | --- | --- | --- | --- | --- | --- |
| ID | ABI-S | PSI-SF | STAI_Y1 | STAI_Y2 | PSDQ_AUTV | PSDQ_AUTN | PSDQ_PERM | MDORS_EMO | MDORS_COSTS | MDORS_INT |
| 1 | 64 | 113 | 55 | 56 | 107 | 46 | 40 | - | - | - |
| 2 | 90 | 130 | 45 | 51 | 104 | 46 | 35 | - | - | - |
| 3 | 78 | 106 | 40 | 37 | 112 | 39 | 25 | - | - | - |
| 4 | 82 | 103 | 35 | 42 | 108 | 44 | 35 | - | - | - |
| 5 | 68 | 112 | 34 | 41 | 104 | 41 | 31 | - | - | - |
| 6 | 84 | 95 | 40 | 62 | 117 | 40 | 30 | - | - | - |
| 7 | 106 | 119 | 36 | 60 | 110 | 34 | 38 | - | - | - |
| 8 | 50 | 127 | 61 | 56 | 112 | 33 | 37 | - | - | - |
| 9 | 82 | 119 | 48 | 58 | 96 | 39 | 35 | - | - | - |
| 10 | 74 | 125 | 40 | 36 | 118 | 39 | 35 | - | - | - |
| 11 | 72 | 129 | 47 | 58 | 94 | 57 | 34 | - | - | - |
| 12 | 64 | 114 | 36 | 47 | 110 | 40 | 34 | - | - | - |
| 13 | 100 | 123 | 44 | 44 | 110 | 33 | 30 | - | - | - |
| 14 | 100 | 140 | 70 | 66 | 114 | 55 | 27 | - | - | - |
| 15 | 112 | 124 | 22 | 45 | 114 | 46 | 31 | - | - | - |
| 16 | 24 | 71 | 21 | 28 | 105 | 46 | 27 | - | - | - |
| 17 | 68 | 100 | 51 | 39 | 109 | 38 | 30 | - | - | - |
| 18 | 36 | 93 | 57 | 74 | 104 | 34 | 32 | - | - | - |
| 19 | 98 | 130 | 52 | 41 | 122 | 36 | 32 | - | - | - |
| 20 | 42 | 93 | 35 | 42 | 104 | 35 | 32 | - | - | - |
| *M* | 74,70 | 113,30 | 43,45 | 49,15 | 108,70 | 41,05 | 32,50 | - | - | - |
| *SD* | 23,14 | 16,44 | 11,91 | 11,39 | 6,70 | 6,60 | 3,72 | - | - | - |
| **3 Months After SD Integration (T1)** | | | | | | | | | | |
| ID | ABI-S | PSI-SF | STAI_Y1 | STAI_Y2 | PSDQ_AUTV | PSDQ_AUTN | PSDQ_PERM | MDORS_EMO | MDORS_COSTS | MDORS_INT |
| 1 | 52 | 110 | 54 | 60 | 109 | 47 | 39 | 36 | 31 | 26 |
| 2 | 74 | 136 | 52 | 45 | 101 | 43 | 31 | 32 | 35 | 29 |
| 3 | 82 | 116 | 59 | 53 | 112 | 33 | 32 | 26 | 40 | 27 |
| 4 | 70 | 109 | 46 | 45 | 109 | 41 | 32 | 45 | 41 | 36 |
| 5 | 46 | 89 | 28 | 37 | 105 | 36 | 24 | 38 | 39 | 29 |
| 6 | 60 | 102 | 51 | 55 | 114 | 45 | 32 | 41 | 44 | 31 |
| 7 | 60 | 92 | 33 | 37 | 116 | 34 | 32 | 42 | 37 | 32 |
| 8 | 62 | 120 | 63 | 53 | 114 | 34 | 37 | 36 | 38 | 27 |
| 9 | 58 | 109 | 42 | 48 | 105 | 40 | 38 | 30 | 44 | 20 |
| 10 | 70 | 124 | 52 | 48 | 118 | 40 | 34 | 38 | 39 | 25 |
| 11 | 64 | 127 | 39 | 56 | 91 | 54 | 34 | 31 | 27 | 17 |
| 12 | 58 | 103 | 26 | 32 | 107 | 35 | 31 | 29 | 37 | 22 |
| 13 | 108 | 125 | 35 | 39 | 111 | 34 | 34 | 42 | 43 | 34 |
| 14 | 120 | 134 | 69 | 69 | 118 | 51 | 28 | 29 | 23 | 21 |
| 15 | 98 | 131 | 46 | 34 | 115 | 41 | 25 | 39 | 41 | 36 |
| 16 | 26 | 62 | 20 | 34 | 116 | 41 | 29 | 43 | 45 | 27 |
| 17 | 40 | 97 | 39 | 30 | 113 | 34 | 27 | 46 | 42 | 30 |
| 18 | 76 | 125 | 74 | 76 | 99 | 38 | 33 | 31 | 34 | 25 |
| 19 | 76 | 99 | 36 | 39 | 115 | 31 | 31 | 49 | 45 | 38 |
| 20 | 36 | 79 | 29 | 35 | 106 | 33 | 27 | 33 | 36 | 25 |
| *M* | 66,80 | 109,45 | 44,65 | 46,25 | 109,70 | 39,25 | 31,50 | 36,80 | 38,05 | 27,85 |
| *SD* | 22,62 | 18,96 | 14,30 | 12,32 | 6,83 | 6,16 | 3,93 | 6,36 | 5,75 | 5,47 |
| **6 Months After SD Integration (T2)** | | | | | | | | | | |
| ID | ABI-S | PSI-SF | STAI_Y1 | STAI_Y2 | PSDQ_AUTV | PSDQ_AUTN | PSDQ_PERM | MDORS_EMO | MDORS_COSTS | MDORS_INT |
| 1 | 42 | 111 | 42 | 46 | 100 | 51 | 38 | 37 | 31 | 29 |
| 2 | 68 | 121 | 44 | 42 | 100 | 44 | 34 | 41 | 36 | 31 |
| 3 | 72 | 118 | 49 | 52 | 108 | 33 | 30 | 28 | 39 | 23 |
| 4 | 68 | 111 | 54 | 50 | 107 | 44 | 38 | 45 | 36 | 31 |
| 5 | 44 | 84 | 32 | 34 | 110 | 37 | 27 | 41 | 37 | 32 |
| 6 | 56 | 77 | 33 | 45 | 118 | 38 | 26 | 40 | 42 | 29 |
| 7 | 56 | 99 | 33 | 40 | 110 | 36 | 35 | 45 | 41 | 37 |
| 8 | 44 | 98 | 60 | 55 | 111 | 32 | 36 | 41 | 42 | 33 |
| 9 | 62 | 107 | 45 | 45 | 105 | 44 | 41 | 41 | 44 | 24 |
| 10 | 60 | 116 | 27 | 31 | 115 | 42 | 33 | 38 | 43 | 24 |
| 11 | 50 | 126 | 30 | 44 | 94 | 56 | 31 | 26 | 28 | 17 |
| 12 | 62 | 97 | 50 | 47 | 120 | 37 | 27 | 33 | 39 | 18 |
| 13 | 104 | 116 | 42 | 42 | 113 | 34 | 33 | 46 | 40 | 36 |
| 14 | 116 | 136 | 68 | 60 | 118 | 59 | 23 | 22 | 17 | 14 |
| 15 | 100 | 137 | 56 | 44 | 115 | 43 | 31 | 40 | 24 | 32 |
| 16 | 28 | 77 | 26 | 28 | 107 | 44 | 29 | 40 | 39 | 24 |
| 17 | 28 | 68 | 26 | 24 | 111 | 31 | 26 | 47 | 43 | 29 |
| 18 | 58 | 104 | 77 | 73 | 112 | 39 | 29 | 22 | 33 | 24 |
| 19 | 70 | 101 | 28 | 28 | 117 | 34 | 25 | 49 | 45 | 38 |
| 20 | 40 | 84 | 26 | 29 | 106 | 33 | 32 | 32 | 38 | 25 |
| *M* | 61,40 | 104,40 | 42,40 | 42,95 | 109,85 | 40,55 | 31,20 | 37,70 | 36,85 | 27,50 |
| *SD* | 22,84 | 18,90 | 14,63 | 11,71 | 6,56 | 7,63 | 4,73 | 7,84 | 6,99 | 6,45 |
| (ID: participant ID; PSDQ_AUTV: Authoritave; PSDQ_AUTN: Authoritarian; PSDQ_PERM: Permissive; MDORS_Emo: perceived emotional closeness; MDORS_Costs: perceived costs; MDORS_Int: child-dog interaction; M: mean score; SD: standard deviation)  *(Recommended clinical threshold cutoff on the total score for the PSI is of≥90; for the STAI-Y1 is of ≥40 and for the STAI-Y2 is of ≥40)* | | | | | | | | | | |

**Supporting Information:
S2 Table. Raw data on the final sample on all standardized scales and questionnaire at each follow-up.**
