# Supplementary material for: Effects of service dogs on children with ASD’s symptoms and parents’ well-being: On the importance of considering those effects with a more systemic perspective
Source: PLoS One. 2024 Jan 3;19(1):e0295702. doi: 10.1371/journal.pone.0295702 (PMC10763939; doi:10.1371/journal.pone.0295702)
Supplement: Attachment — Submitted filename: Response to Reviewers.docx [file pone.0295702.s003.docx]

**Rebuttal letter**

Journal PLOS ONE (eISSN 1932-6203)

Manuscript ID PONE-D-23-30617

Type Article

Title : Effects of service dogs on children with ASD’s symptoms and parents' wellbeing: on the importance of considering those effects with a more systemic perspective.

Authors: Nicolas Dollion^*^, Margot Poirier, Association Handi’Chiens, Fondation Mira, Florian Auffret, Nathe François, Pierrich Plusquellec, Marine Grandgeorge.

**Academic Editor Notes**

Thank you for submitting your manuscript to PLOS ONE. After careful consideration, we feel that it has merit but does not fully meet PLOS ONE’s publication criteria as it currently stands. Therefore, we invite you to submit a revised version of the manuscript that addresses the points raised during the review process.

The two reviewers addressed several major concerns about your manuscript. Please revise your manuscript according to reviewer's comments.

*We would like to thank the academic editor and both reviewers for their important and constructive comments. We tried our best to address all comments and comply to Plos One’s requirements for manuscript formatting.*

Please include the following items when submitting your revised manuscript:

- A rebuttal letter that responds to each point raised by the academic editor and reviewer(s). You should upload this letter as a separate file labeled 'Response to Reviewers'.
- A marked-up copy of your manuscript that highlights changes made to the original version. You should upload this as a separate file labeled 'Revised Manuscript with Track Changes'.
- An unmarked version of your revised paper without tracked changes. You should upload this as a separate file labeled 'Manuscript'.

In addition to the present rebuttal letter, two versions of the manuscript have been generated and submitted on the submission platform: one corresponding to the revised version with all changes made highlighted in yellow ('Revised Manuscript with Track Changes'); the second corresponding to the unmarked revised version (‘Manuscript’).

If you would like to make changes to your financial disclosure, please include your updated statement in your cover letter. Guidelines for resubmitting your figure files are available below the reviewer comments at the end of this letter.

No changes to our initial financial disclosure had to be performed.

If applicable, we recommend that you deposit your laboratory protocols in protocols.io to enhance the reproducibility of your results. Protocols.io assigns your protocol its own identifier (DOI) so that it can be cited independently in the future. For instructions see: <https://journals.plos.org/plosone/s/submission-guidelines#loc-laboratory-protocols>. Additionally, PLOS ONE offers an option for publishing peer-reviewed Lab Protocol articles, which describe protocols hosted on protocols.io. Read more information on sharing protocols at <https://plos.org/protocols?utm_medium=editorial-email&utm_source=authorletters&utm_campaign=protocols>.

Not applicable.

Journal Requirements:

When submitting your revision, we need you to address these additional requirements.

1. Please ensure that your manuscript meets PLOS ONE's style requirements, including those for file naming. The PLOS ONE style templates can be found at 
https://journals.plos.org/plosone/s/file?id=wjVg/PLOSOne_formatting_sample_main_body.pdf and 
<https://journals.plos.org/plosone/s/file?id=ba62/PLOSOne_formatting_sample_title_authors_affiliations.pdf>

*We ensured that the revised manuscript meets all Plos One’s style requirements, and that file naming respects Plos One’s guidelines.*

2. In your Data Availability statement, you have not specified where the minimal data set underlying the results described in your manuscript can be found. PLOS defines a study's minimal data set as the underlying data used to reach the conclusions drawn in the manuscript and any additional data required to replicate the reported study findings in their entirety. All PLOS journals require that the minimal data set be made fully available. For more information about our data policy, please see http://journals.plos.org/plosone/s/data-availability.

Upon re-submitting your revised manuscript, please upload your study’s minimal underlying data set as either Supporting Information files or to a stable, public repository and include the relevant URLs, DOIs, or accession numbers within your revised cover letter. For a list of acceptable repositories, please see http://journals.plos.org/plosone/s/data-availability#loc-recommended-repositories. Any potentially identifying patient information must be fully anonymized.

*Our study’s minimal underlying data set was previously directly embedded with the manuscript as Supplementary material Table B. In this revised version of the manuscript, the minimal data has been integrated as Supporting Information in a separate file (S2 Table).*

3. Please upload a copy of Supporting Information Figure/Table/etc. Supplementary Material: Table A and Table B which you refer to in your text on pages 46 and 47.

*In the revised version of the manuscript, Table A and B are integrated as Supporting Information (S1 Table and S2 Table) in separate dedicated files.*

**Reviewers' comments:**

1. Is the manuscript technically sound, and do the data support the conclusions?
Reviewer #1: Partly
Reviewer #2: Partly

*No adjustment requested.*

2. Has the statistical analysis been performed appropriately and rigorously?
Reviewer #1: I Don't Know
Reviewer #2: N/A

*No adjustment requested.*

3. Have the authors made all data underlying the findings in their manuscript fully available?
Reviewer #1: Yes
Reviewer #2: Yes

*No adjustment requested.*

4. Is the manuscript presented in an intelligible fashion and written in standard English?
Reviewer #1: No
Reviewer #2: Yes

*English has been revised by an English native professional.*

5. Review Comments to the Author
Reviewer #1: No specific comments to the authors. See my letter below to the editor.
xxxx xxxx xxxx xxxxx xxxx xxxx
wwwwwwwwwwwwwwwwwwwww
eeeeeeeeeeeeeeeeeeeeeeeeeee
rrrrrrrrrrrrrrrrrrrrrrrrrrrrrrrrrrrr
111111111111111111111111111
222222222222222222222222222
333333333333333333333333333
4444444444444444444444444444
555555555555555555555555555555
66666666666666666666666666666666
88888888888888888888888888888
99999999999999999999999999999
111111111111111111111111111111111111111111111111111111111111111111111
222222222222222222222222222222222222222222222222222222222222222222222
333333333333333333333333333333333333333333333333333333333333333333333333333
444444444444444444444444444444444444444444444444444444444444444444444444
555555555555555555555555555555555555555555555555555555555555555555555555
666666666666666666666666666666666666666666666666666666666666666666666666
777777777777777777777777777777777777777777777777777777777777777777777777
888888888888888888888888888888888888888888888888888888888888888888888

*No adjustment requested.*

Reviewer #2: The authors stated that 'To the best of our knowledge, this study is the first to quantitatively investigate the reciprocal influences between the benefits of SD (Service Dog) integration on children with ASD (Autism spectrum disorder) and their parents, as well as the first to explore the association of these benefits with parenting strategies and the child-SD relationship, using a longitudinal approach. '

This study is very interesting. However I have a few questions.

#1: Would you tell me the definition of parenting strategies in this study?

*We thank reviewer #2 for this comment. “Parenting strategies” was not clearly defined in the initial version of the manuscript. Additionally, this comment allowed us to notice that in the initial version of the manuscript sometimes the term “parenting profile” was used and other times “parenting style” was used (which in fact are common expressions referring to the same concept in the scientific literature), which could have increased the confusion to the reader on the difference between parenting style and parenting strategies.*

*The definition of parenting strategies is now clearly stated, and only the expression “parenting style” used. In line with those modifications, we also adjusted the presentation of the PSDQ.*

*P.4-5. “**Parenting strategies correspond to behaviors, attitudes and practices sharing common traits that parents may use and express when they communicate and interact with their child. Based on their tendency to rely on specific parenting strategies rather than others, parents’ parenting style can be drawn [39]. Parenting styles can notably be defined according to two main parenting dimensions (i.e., warmth and control), and four main parenting styles can be distinguished based on their parents’ positioning on those dimensions: Authoritative [warmth+/control+], Authoritarian [warmth-/control+], Permissive [warmth+/control-] and Disengaged [warmth-/control-]* *[40-43].* *~~The a~~Authoritative parenting strategies and style are ~~is~~ generally considered to lead to more positive developmental outcomes for the child, compared to other parenting strategies and style, such as for example, authoritarian parenting, which may lead to greater internalizing behaviors in children [44]. […] In terms of parenting styles, the authoritarian style has been shown to be prevalent in parents of children with ASD compared to parents of NT children, while they rely less on authoritative strategies [27,54,55]. Interestingly, the higher level of stress experienced by parents of children with ASD’s appears to contribute to these differences in parenting strategies. In their study, Boonen and collaborators [51] demonstrated that while parents of children with ASD showed differences in their parenting behaviors compared to parents of NT children (i.e., lower scores on sensitivity and higher scores on material rewarding), these differences did not remain significant when statistically controlling for the effect of parenting stress.”*

*P.14 . “The PSDQ [94,95] is a 62-item* *questionnaire designed to assess parent’s childrearing behavior and strategies according to the three major parenting styles defined by Baumrind [40]: authoritative (i.e., warm/involved, reason/induce, democratic participation, good nature/easygoing), authoritarian (i.e., verbally hostile, uses corporal punishment, does not reason, uses punitive strategies, directive) and permissive (i.e., lacks follow-through, ignores misbehavior, and lacks self-confidence).”*

#2: Related to #1, has a psychiatric evaluation been done on the parents?

*We did not perform a psychiatric evaluation on parents. However, it would have indeed been of interest in order to draw additional conclusion; notably to explore if parent’s depression and anxiety disorder is related to variables prior to SD integration (i.e., child’s ASD symptoms, parenting strategies), and to changes after SD integration. We added this element in the “****5. Limits and future directions” section (third paragraph)****:*

*P.33-34 “Due to its multi-dimensional nature, wellbeing is a complex concept that encompass numerous elements, such as feeling of competence, self-esteem, positive emotion and resilience. However, in the present study, effect of service on parental well-being parental was considered only through its on parental anxiety and parenting stress. It would thus be of interest for future studies to consider extending investigation of the effects of SD on other aspects of well-being and/or to use multidimensional measures of well-being, such as developed by Huppert and So [107].* *Additionally, performing a psychiatric evaluation on parents prior to the integration of the SD, could have been of interest. Indeed, knowing parents of children with ASD are at higher risks of psychological health issues, notably anxiety and depression, compared to parents of NT children [24-27], it would have been of interest to perform such type of evaluation in order to explore if parents initial mental health status was related to their child with ASD’s symptoms and to their parenting strategies, as well as with the different benefits and changes observed after SD integration.”*
